# Supplementary material for: The Contribution of Postprandial Glucose Levels to Hyperglycemia in Type 2 Diabetes Calculated from Continuous Glucose Monitoring Data: Real World Evidence from the DIALECT-2 Cohort
Source: Nutrients. 2024 Oct 20;16(20):3557. doi: 10.3390/nu16203557 (PMC11510104; doi:10.3390/nu16203557)
Supplement: Supplementary file 1 [file nutrients-16-03557-s001.zip › nutrients-3225338-supplementary.pdf]

Supplementary File S1 Assumptions used to calculate compliance for breakfast, lunch, and dinner based on food records filled in by patients.

1. **Recording Food Intake**: Patients were instructed to record the quantity of food intake for each item in grams, household measures, or natural units. When this information was missing, compliance for that particular food item was considered as zero.
    a. For a wide range of household measures and natural units, we employed commonly used measurements in grams per day.
    b. Reported quantities such as 'a portion' or 'a plate' were deemed insufficiently specific and were thus regarded as zero.
    c. Reported quantities expressed as '1 beverage' lacked specificity and were also recorded as zero. It should be reported in terms of a cup, glass, or mug.
    d. For product-specific data, such as the source of bread and additions to coffee (milk and sugar), assumptions were made based on previous days' data. When a patient reported such product-specific data for at least three days, it was assumed to apply for the entire period.
2. **Bread-based meals:** For meals centered around bread (typical lunch in the Netherlands), patients were required to report the number of bread slices, the number of spreads, and one beverage. Compliance was considered as 100% when at least two elements were reported with a quantity.
    a. Reported quantities such as '1 slice of bread with cheese' were assumed to represent one slice of bread with one slice of cheese in a 1:1 ratio. When a patient reported 4 slices of bread with 1 slice of cheese and 'jam,' it was assumed that the amount of 'jam' was 3.
    b. Reported quantities as 'a double sandwich' were assumed to be two slices of bread in a 1:2 ratio.
    c. Quantities of bread were assumed to be in terms of slices of bread. Bread rolls were assumed to be soft bread rolls unless specified otherwise.
    d. Many luxury bread rolls did not require spreads, such as currant buns, pizza bread, sausage rolls, croissants, etc. For these types of bread, the component related to the number of spreads was disregarded, and the component concerning the amount of bread remained.
    e. In cases where the type of bread was not reported, it was assumed to be brown bread.
3. **Yoghurt-based meals:** For meals centered around yogurt, patients were required to report the quantity of yogurt, the number of grains (e.g., oatmeal, muesli), and one beverage. Compliance was considered as 100% when at least two elements were reported with a quantity.
    a. When the ratio was known based on data from at least three previous days, this ratio was used to calculate the number of grains relative to the current amount of yogurt. For example, if 250 ml of yogurt equalled 50 grams of grains, a 5:1 ratio was applied for other quantities of yogurt.
    b. In cases where the type of yogurt was not reported, it was assumed to be semi-skimmed yogurt.
4. **Hot Meals:** For hot meals, patients were instructed to report the quantity of starchy products (e.g., potatoes, pasta, rice), the quantity of meat, poultry or fish, the quantity of vegetables, and a type of sauce (gravy, pasta sauce, or frying fat) or dessert. Compliance was considered as 100% when at least three elements were reported with a quantity.
    a. For soups, it was necessary to specify the content in terms of meat, vegetables, and noodles, except for cup-a-soup.
    b. Potato products were required to be reported in terms of boiled, baked, or mashed potatoes, along with the corresponding serving size.
    c. Reported quantities such as '4 serving spoons hotchpot' were assumed to represent a 1:1 ratio of 2 serving spoons of boiled potatoes and 2 serving spoons of vegetables.

d. Many luxury dinners did not require all elements, such as pancakes, pizza, fries, gourmet, barbecue, etc. These meal types were considered compliant if at least 50% of reported amounts were provided.

5. **Multiple Meals:** In cases of multiple meals, the contents were aggregated. For example, if a patient consumed one bowl of hot porridge together with a slice of bread with cheese for breakfast, five components were expected to be reported: the quantity of bread slices, the quantity of cheese slices, the quantity of oatmeal, the quantity of yogurt, and one beverage. At least 50% compliance was required, which in this case equated to reporting a minimum of three quantities.
